# Quality of life and pain in patients with metastatic bone disease from solid tumors treated with bone-targeted agents– a real-world cross-sectional study from Switzerland (SAKK 95/16)

**DOI:** 10.1186/s12885-021-07903-8

**Published:** 2021-02-19

**Authors:** Karin Ribi, Beat Thürlimann, Corinne Schär, Daniel Dietrich, Richard Cathomas, Ursina Zürrer-Härdi, Thomas von Briel, Sandro Anchisi, Pierre Bohanes, Veronika Blum, Philippe von Burg, Meinrad Mannhart, Clemens B. Caspar, Roger von Moos, Michael Mark

**Affiliations:** 1 International Breast Cancer Study Group (IBCSG), Bern, Switzerland; 2 Kantonsspital St. Gallen, St. Gallen, Switzerland; 3 Swiss Group for Clinical Cancer Research (SAKK) Coordinating Center, Bern, Switzerland; 4 Kantonsspital Graubünden, Chur, Switzerland; 5 Kantonsspital Winterthur, Winterthur, Switzerland; 6 Klinik Hirslanden, Zürich, Switzerland; 7 Hôpital du Valais, Sion, Switzerland; 8 Centre de Chimiothérapie Anti-Cancéreuse, Lausanne, Switzerland; 9 Kantonsspital Luzern, Luzern, Switzerland; 10 Burgerspital Solothurn, Solothurn, Switzerland; 11 Andreasklinik Cham Zug, Cham, Switzerland; 12 Kantonsspital Baden, Baden, Switzerland

**Keywords:** Bone metastases, Bone-targeting agents, Patient-reported pain, Bone-pain related quality of life, Non-interventional, Patterns of care

## Abstract

**Background:**

Bone-targeted agents (BTAs) are widely used in the management of patients with bone metastases from solid tumors. Knowledge of the impact of their routine care use on patient-reported pain and bone pain-related quality of life (QoL) is limited.

**Methods:**

This real world, cross-sectional study enrolled patients over a 3-month period through oncologists across Switzerland. Patients were ≥ 18 years, had solid tumors and at least one bone metastasis, and received routine care for bone metastases. Physicians provided data on BTA-related practices, risk of bone complications and BTA regimen. Patients completed questionnaires about pain (BPI-SF), general and bone pain-related QoL (FACT-G, FACT-BP) and treatment satisfaction (FACIT-TS-G).

**Results:**

Eighteen sites recruited 417 patients. Based on the FACT-BP, 42% of the patients indicated not having bone pain. According to the BPI-SF, 28% reported no, 43% mild, 14% moderate, and 15% severe pain, respectively. Patients not treated with a BTA had better overall QoL (FACT-G: *p* = 0.031) and bone pain-related QoL (FACT-BP, *p* = 0.007) than those treated with a BTA. All pain and other QoL scales did not differ between groups. Patients perceived at ‘low risk of bone complications’ by their physician not receiving a BTA reported less pain and better QoL than those considered at ‘low risk’ but receiving BTA treatment or those considered at ‘high risk’ regardless of BTA treatment. Overall satisfaction with the treatment was good; almost 50% of patients reporting that they were completely satisfied.

**Conclusions:**

Overall, pain and QoL did not differ according to BTA treatment or physicians’ risk perception. Patient with low risks not receiving BTA treatment reported least pain and highest QoL scores. These results may suggest that treating physicians assess bone complication risk appropriately and treat patients accordingly, but they need to be confirmed by objective determination of longitudinal skeletal complication risk.

**Supplementary Information:**

The online version contains supplementary material available at 10.1186/s12885-021-07903-8.

## Background

Bone metastases are common in patients with solid tumors and are frequently associated with skeletal complications, known as skeletal-related events (SREs) and symptomatic skeletal events (SSEs) [1]. An increase in bone pain at the onset and diagnosis of bone metastases often leads to a decrease in quality of life (QoL) [2–5]. Further symptoms including fatigue, trouble sleeping, numbness and tingling can also follow within 1 year after diagnosis [3]. Bone-targeted agents (BTAs) are widely used in clinical practice to delay the onset of SREs and bone pain, and thereby to maintain or delay a decrease in QoL [1, 6].

The effect of BTAs on QoL has mainly been evaluated by comparing the RANK ligand inhibitor denosumab with bisphosphonates. In a pooled analysis of three double blind randomized clinical trials (RCTs) with patients with solid tumors (breast, prostate and others), clinically meaningful improvements in QoL were similar for both agents [7]. However, fewer patients experienced a clinically meaningful worsening in QoL when treated with denosumab compared to bisphosphonates. The recent REaCT-BTA trial compared the effect of 12- versus 4-weekly BTA regimens for 1 year on QoL in patients with bone metastases from breast and prostate cancer [8]. Results showed no statistically significant differences between the 12- and 4-weekly dosing regimens in any of the evaluated QoL outcomes [8]. Due to limitation with regard to sample size, the clinical relevance of these findings remains unclear. In patients with non-small cell lung cancer, the evidence that BTAs prevent pain or have a favorable impact on QoL is still weak [2].

In addition to the ongoing collection of robust data on the long-term safety and QoL impact of BTA therapy in randomized clinical trials, data from observational studies on current practices of BTA use in routine care have been published [9–11]. A study conducted across six European countries in patients with advanced breast cancer [9] and patients with prostate cancer [10], showed that patients with bone metastases reported worse pain and QoL than those without bone metastases. In the subgroup of patients with advanced breast cancer who had bone metastases, those receiving a BTA reported significantly lower average pain severity scores and lower scores for pain interference with daily activities than those who did not receive a BTA [9]. This study used a well-known, validated, general measure for pain and QoL. An instrument validated for bone metastases-related QoL including questions specific to bone pain may provide additional understanding of patients’ expectations and acceptance of bone-specific therapies [12]. In addition to patient-reported outcomes (PROs), so-called patient-reported experiences (PREs) gain importance as indicators for the quality of care. PREs include patient’s needs, experiences and satisfaction whilst receiving care [13]. The patient view is important as a prolonged use of BTAs may raise concerns about additional benefit, cumulative risks for adverse events (e.g., osteonecrosis of the jaw), treatment costs and inconvenience for patients [14].

A recent multicenter, observational study provided real-world insight into the routine care of BTA prescribing practices of physicians treating patients with bone metastases from solid tumors in Switzerland [11]. PROs were also collected, including pain, and bone-metastases-related QoL and satisfaction with treatment. Variation in BTA administrations may results in different patient experiences. We aimed to understand the impact of BTA treatment on patient-reported pain, QoL, and satisfaction with treatment by taking physicians’ estimation of risk for bone complications into account.

## Methods

### Study design

This cross-sectional observational survey study aimed at obtaining information on the real-world use or non-use of BTAs and their effect on patients’ bone pain, general and bone-pain-related QoL in Switzerland. Participating physicians all over Switzerland documented every patient with bone metastases coming for a regular visit during a given time period of 3 months. This allowed capturing all patients with bone metastases independent from BTA treatment and BTA administration interval. The study was approved by the cantonal ethics committee of Zurich BASEC 2017–01337 for all participating sites.

### Physicians and patients

Physicians participating in the study were identified via the Swiss Group for Clinical Cancer Research network supported by the Swiss medical oncology association. They could practice at either public hospitals or private clinics/practices and had to be personally responsible for patient treatment decisions at their center. The physicians who agreed to participate identified patients under their treatment. Patients were eligible if they were aged ≥18 years; had solid tumors and at least one bone metastasis. They had to attend regular visits during the 3 months the physician’s center was participating in the study. All patients provided written informed consent before participating.

Physicians had to provide details on their clinical context (e.g., specialism, experience, type of center, caseload) and BTA prescribing behaviors (preferred agent, dosing schedule, factors that influence BTA-related clinical decision-making) [11]. They also completed a study-specific questionnaire for each eligible patient regarding patient characteristics, BTA use, preferred agent, dosing frequency and clinical drivers of BTA initiation, risk of bone complications (i.e., at high risk of pathological fractures, surgery or radiation to bone, or spinal cord compression) and related outcomes (bone complication incidence, bone pain and analgesia use) (Table S1). The risk perception was based on the physician’s clinical opinion.

### Assessments

Patients assessed their pain severity with the Brief Pain Inventory – Short Form (BPI-SF) at the time of their visit. The BPI-SF is a well-validated and commonly used self-report measure to assess pain severity and pain interference with daily activities and QoL [15]. In this study, we only assessed pain severity (“worst”, “least”, “average”, “right now”). Patients rated their pain for each scale from 0 (no pain) to 10 (pain as bad as you can imagine). Patients’ worst pain scores were classified as follows: 0 for no pain, 1 to 4 for mild pain, 5 or 6 for moderate pain, and 7 to 10 for severe pain [16, 17]. Worst-pain severity ratings also were dichotomized into a no or mild pain category (score 0–4) and a moderate or severe pain category (score 5–10).

In addition, patients completed a cancer-specific QoL questionnaire, the Functional Assessment of Cancer Therapy – General (FACT-G), and a bone-pain module, the FACT-BP [18]. The FACT-G covers four subscales: physical, social/ family, emotional, and functional well-being. Patients rate how they have felt over the past 7 days, on a scale of 0 (“not at all”) to 4 (“very much”). Each subscale is scored 0–28 (except for the emotional well-being subscale 0–24). The FACT-G total score can be calculated by adding up the four subscales (score range 0–108). The FACT-BP is a 16-item scale assessing cancer-related bone pain and its effects on QoL in the same response format as the FACT-G (score range 0–60). Higher scores indicate less bone pain and/or better QoL.

To evaluate expectations and satisfaction with treatment, patients further completed the FACIT-TS-G (Functional Assessment of Chronic Illness and Treatment – Treatment Satisfaction-General) [19]. Patients were instructed to answer the questions by considering specifically their BTA treatment. The eight single items assess patients’ perception of effectiveness and side-effects of treatment, physician support, recommending the treatment to others, decision making and overall treatment evaluation. The response format varies according to question type.

### Statistical considerations

As this is a cross-sectional descriptive study, no formal sample size calculation was performed. All eligible patients were included in the analysis. Categorical variables are reported as frequencies and percentages. Continuous variables include the total scores, subscales and single items of the FACT-G, FACT-BP, FACIT-TS-G and BPI-SF [15, 20]. Differences between groups were tested by Wilcoxon–Mann–Whitney tests or Kruskal-Wallis tests. A difference of ≥3 points in the FACT-BP [18] and ≥ 4 in the FACT-G [21] is considered clinically relevant. McNemar’s test of symmetry tested the accordance between physicians and patients’ pain ratings. Analyses were performed with SAS 9.4 (SAS Institute Inc., Cary, NC, USA).

## Results

Between November 2017 and May 2018, 86 oncologists from 18 sites across Switzerland participated in the study. The 3-month period of participation varied for each center, resulting in a longer than 3 months overall study period. Eighty percent (*N* = 69) of the participating physicians reported working in public hospitals and 20% (*N* = 17) in private institutions. Almost half (48%) had 10–20 years’ medical expertise, 20% had between 5 and 10 years’ experience, 17% had ≤5 years’ experience, followed by 15% with > 20 years’ medical expertise.

### Patient and disease characteristics

The 18 sites recruited 417 patients with advanced solid tumors and bone metastases. The most common underlying tumor type was breast cancer, followed by prostate and lung cancer (Table 1). The majority of patients showed disease stabilization at the time of assessment. Approximately two thirds of patients were under active antitumor treatment (hormone therapy, chemotherapy and/or radiotherapy). More than three out of four patients (79%) had at least three bone metastases. The most common sites of bone metastases were vertebrae locations (75%) and in the hip/pelvis (66%) (Table 1). The vast majority of the patients (92%) was not experiencing bone complications at the study entry assessment. The frequency of co-morbidities was collected at study start, with the most common (> 8%) conditions reported for patients being hypertension (38%), diabetes mellitus (10%), chronic obstructive pulmonary disease (9%), renal impairment (9%) and coronary heart disease (8%). Half of the patients were 69 years or older.

**Table 1:** Patient demographics and clinical characteristics (*N* = 417 patients)

Median age (min., max.)	69 (30, 95)
**Underlying tumor entity**	**N (%)**
Breast cancer	169 (41)
Prostate cancer	106 (25)
Lung cancer	62 (15)
Other	80 (19)
**Disease status**	**N (%)**
Progressing	112 (27)
Stable	302 (72)
Missing	3 (1)
**ECOG performance status**	**N (%)**
0	140 (34)
1	210 (50)
2	53 (13)
3	14 (3
**Cancer treatments (multiple)**	**N (%)**
Chemotherapy	258 (62)
Endocrine therapy	264 (63)
Targeted treatments	100 (24)
Immunotherapy	82 (20)
Radiotherapy	251 (60)
Radioisotope therapy	34 (8)
Surgery	207 (50)
Unknown	6 (1)
**Employment status**	**N (%)**
Working part time	67 (16)
Working full time	38 (9)
Unemployed	46 (11)
Retired	261 (63)
Unknown	5 (1)

### Patient-reported pain and bone pain-related QoL

At the time of assessment, 28% of the patients (112/400) with available BPI-SF scores were not experiencing pain; 43% (174/400) reported mild pain, 14% (55/400) had moderate and 15% (59/400) had severe pain. According to the FACT-BP single-item (Table S2), 42% of the patients indicated not having bone pain. A considerable proportion of patients (44 to 78% depending on question) indicated that bone pain did “not at all” affect physical, social or emotional aspects of their life. Patients who indicated to have no or mild pain (BPI-SF ≤ 4) reported better scores for all QoL scales (Table S3).

While 114 (29%) patients experienced moderate-to-severe pain (BPI-SF ≥ 5), physicians reported moderate to severe pain in 62 (15%) patients (Table S4). Patients’ rated their pain as more severe than physicians (*p* < 0.001).

### Patient-reported pain and analgesic use

**Fig. 1:**
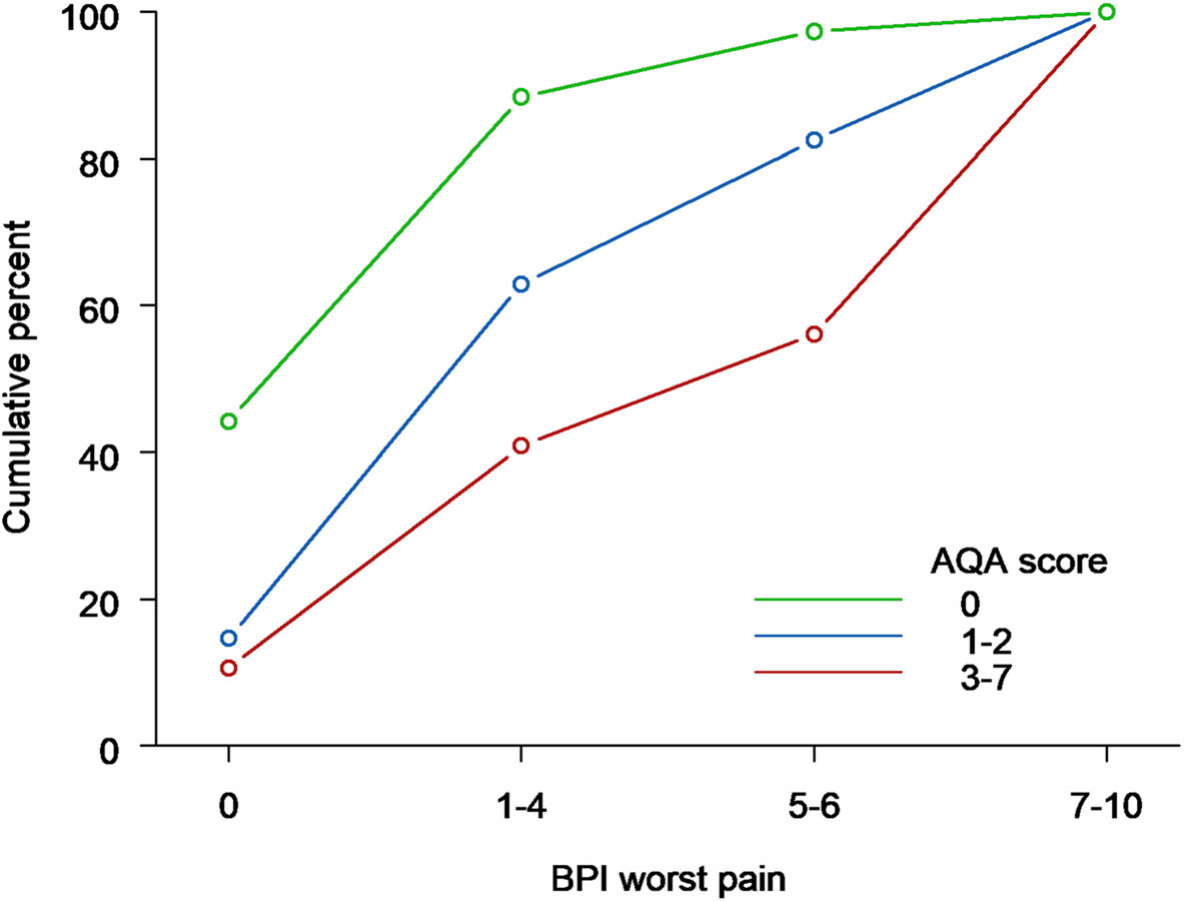
Distribution of BPI worst pain by Analgesic Quantification Algorithm (AQA) score

Among patients with available BPI-SF and Analgesic Quantification Algorithm (AQA) scores, approximately half of the patients (190/399, 48%) were not receiving analgesics, 33% (133/399) were receiving non-opioid analgesics and 17% (66/399) strong opioids. In the majority of patients, analgesic use corresponded with their pain reports (Fig. 1, Table S5). Among patients without pain, 44% were not receiving analgesics, among those with mild-to-moderate pain, 68% were receiving non-opioid analgesics, and among those with severe pain, 44% were receiving strong opioids.

### Pain and QoL by BTA treatment and risk for bone complications

Mean BPI-SF pain severity did not significantly differ between patients who received BTA treatment compared to those who did not (Table 2). When looking at pain severity groups, patients with mild pain were more often not treated than treated with BTA. For the other severity groups, there was no apparent association between pain severity and BTA treatment (Table 3). FACT-G subscales scores for physical, social, emotional and functional wellbeing were similar in BTA-treated and untreated patients. However, patients who were not treated with a BTA reported better overall QoL (FACT-G mean difference: 4, 95% CI: 0.33, 7.5; *p* = 0.031) and bone pain-related QoL (FACT-BP mean difference: 3, 95% CI: 0.3, 4.0; *p* = 0.007) compared to patients treated with a BTA (Table 2).

**Table 2:** Patient-reported outcomes by BTA treatment and risk status

	Patients treated with BTA therapy			Patients not treated with BTA therapy				High bone complication risk			Low bone complication risk			
	N	Mean	SD	N	Mean	SD	***p*** value^**3**^	N	Mean	SD	N	Mean	SD	***p*** value^**3**^
**Pain (BPI-SF)**^a^														
Worst pain	296	3.1	2.9	104	2.5	2.7	0.076	229	3.0	2.9	155	2.9	2.7	0.684
Least pain	295	1.2	1.6	104	1.1	1.6	0.815	228	1.1	1.6	155	1.2	1.6	0.364
Average pain	296	2.1	2.1	104	1.9	2.1	0.249	229	2.1	2.1	155	2.0	2.0	0.957
Pain right now	296	1.7	2.2	104	1.4	1.9	0.359	229	1.5	2.1	155	1.7	2.1	0.414
**Bone pain (FACT-BP)**^b^	301	47.7	12.4	105	50.7	11.0	0.007	230	48.1	12.1	160	48.9	11.9	0.397
**Quality of Life (FACT-G)**^b^														
Physical wellbeing	302	20.5	5.7	105	21.5	5.4	0.084	231	20.6	5.8	160	20.8	5.6	0.693
Social/family wellbeing	299	21.7	5.0	105	22.6	4.8	0.101	231	21.9	5.0	158	22.0	4.8	0.961
Emotional wellbeing	300	17.4	4.7	105	18.5	4.2	0.060	231	17.7	4.7	158	17.6	4.5	0.698
Functional wellbeing	303	17.8	5.4	105	18.9	5.2	0.078	232	17.7	5.5	160	18.5	5.0	0.205
FACT-G total score	296	77.4	15.5	105	81.4	14.4	0.031	229	78.1	15.4	157	78.8	15.5	0.665

^a^ Higher score indicate worse pain

^b^ Higher scores indicate less bone pain or better QoL

^3^ Univariate Wilcoxon–Mann–Whitney tests

**Table 3:** BPI-SF pain scores according to BTA treatment and risk status^a^

	Overall sample Receiving BTA Therapy		Known risk status Bone complication risk	
	No (***n*** = 104)	Yes (***n*** = 296)	High (***n*** = 229)	Low (***n*** = 155)
**BPI-SF Worst pain**	n (%)	n (%)	n (%)	n (%)
No	31 (30)	81 (27)	64 (28.0)	43 (28)
Mild	53 (51)	121 (41)	97 (42.7)	71 (46)
Moderate	8 (8)	47 (16)	34 (14.5)	19 (12)
Severe	12 (11)	47 (16)	34 (14.5)	22 (14)

Note: Pain assessment was missing for 17 patients;

^a^ risk of bone complications was defined by the treating physician based on estimated risk for pathological fractures, surgery or radiation to bone, or spinal cord compression

**Fig. 2:**
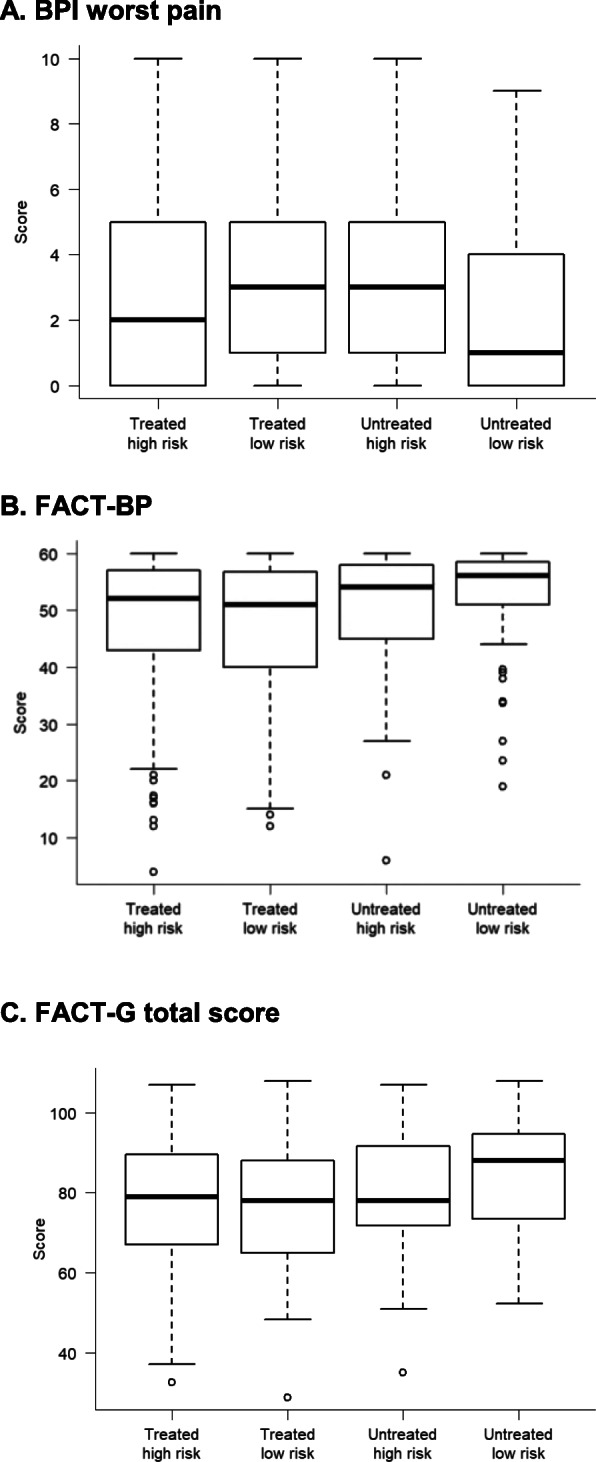
Boxplots for pain and QoL by BTA treatment (yes/no) and risk status (low /high). **a** BPI worst pain; **b** FACT-BP, FACT-G total score. Higher scores for the BPI worst pain indicated worse pain; higher scores for the FACT-BP and FACT-G indicate better QoL

More than half of the patients (235/417, 56%) were considered to be at high risk of bone complications by their treating physicians. No association between pain or any of the QoL outcomes and perceived risk of bone complications (i.e., high- vs. low-risk patients as categorized by their treating physicians) were observed (Table 3). Patients considered at ‘low risk of bone complications’ not receiving a BTA reported significantly lower ‘worst pain’ scores (*p* = 0.025) and better bone pain-related QoL scores (*p* = 0.012) than those considered at ‘low risk’ but receiving BTA treatment or those considered at ‘high risk’ regardless of BTA treatment (Fig. 2, Table S6).

### Patients’ expectations and satisfaction with treatment

The majority of patients (80%) rated the effect of BTA treatment as “good” to “excellent” (Table S7). More than half of the patients (55%) rated the effectiveness of the BTA treatment and 41% the side effects of treatment as better than expected. The majority also felt that their doctor did help them evaluate the treatment effects (78%), and that this was the right treatment for them (83%). Overall satisfaction with the treatment was good, with almost 50% of patients being completely satisfied. Over 60% of the patients would recommend the treatment to someone with the same condition and would choose it again.

## Discussion

This study provides complementary data on patient-reported pain and bone pain-related QoL from a multicenter, observational study about BTA prescribing practices of physicians treating patients with bone metastases from solid tumors [11]. Patients reported on average mild pain, 71% of all patients and 81% of patients not receiving a BTA, respectively, reported no or mild pain. When asked specifically for bone pain, 42% reported no pain. Between 44 and 78% indicated that bone pain did “not at all” interfere with different aspects of daily life. However, patients who reported moderate-to-severe pain (BPI-SF) had worse QoL scores than those who reported no or mild pain confirming results from previous studies showing a negative impact of pain on QoL [1].

In comparison, a pooled analysis based on individual patient data from three RTCs showed no or mild pain in 53% of patients with solid tumors and bone metastases before starting treatment with denosumab or zoledronic acid [7]. Average QoL scores were worse than were those observed in our study. The comparability of our results to those of RCTs is limited. RCTs focused on changes in pain and QoL during BTA treatment and included patients who were early in the disease course while our results reflect the situation of patients at different stages of their disease. Pain and QoL scores in our study also compare favorably to those observed in a cross-sectional real-world study for patients with advanced breast [9] or prostate cancer [10] who presented with bone metastases.

Data from our study indicates that treating physicians achieve an adequate pain management for their patients. The majority of patients in each pain severity group received analgesics corresponding to their pain reports whereas data from another real-world study suggests under-treatment of pain in patients with prostate cancer and bone metastases [10]. Patients in our study probably received a good overall disease management with effective anti-cancer treatments and supportive care measures, including adequate pain and hypocalcemia management. Switzerland is a wealthy country providing universal and rapid access to the health care system including treatments for malignant osteolytic bone disease.

The comparison of patients who were treated to those who were not treated with a BTA showed no differences between these groups for pain severity or for physical, social, emotional and functional wellbeing. Interestingly, patients not treated with a BTA reported clinically relevant better overall and bone pain-related QoL than patients treated with a BTA. In contrast, existing real-world practice data indicated that advanced breast cancer patients who were receiving a BTA presented with significantly lower average pain severity than those not receiving a BTA [9]. Moreover, one of the early studies on BTA treatment effects in patients with breast cancer and lytic bone metastases showed significantly less increase in bone pain with bisphosphonate pamidronate disodium compared to placebo [22].

According to the physicians in our study, ‘high risk’ of bone complications and bone pain were the most common drivers of BTA initiation [11]. Physicians’ risk perception alone was not associated with any of the PROs. Patients perceived at ‘low risk’ of bone complications not receiving a BTA were those reporting less pain and better QoL than those considered at ‘low risk’ who did receive BTA treatment or those considered at ‘high risk’ regardless of BTA treatment. Differences in QoL between patients with ‘high’ and ‘low’ risks for bone complications may be a consequence of varying disease burden. Differences between physicians in terms of risk perception were not taken into account.

Among patients who received a BTA almost 50% of patients reported complete satisfaction with treatment, 80% perceived the effect of BTA treatment as “good” to “excellent”. These estimations underpin the appropriate pain management and low incidence of SREs observed in this population most likely due to the relatively high proportion (71%) of physicians reporting a guideline-recommended BTA prescription [11]. A recent retrospective study among German oncologists found guideline adherence to be associated with bone pain improvement in lung and breast cancer patients [23]. Overall, our results are indicative for the ability of treating physicians using their clinical judgement to assess bone complication risk and to treat appropriately. Although guidelines based on RCTs apply in general, the individual patient requires considerations on whether at all using a BTA, and, if yes, on the optimal dose, start and duration. No randomised data is available to guide whether all patients with bone metastases should initiate a BTA as soon as bone metastases are diagnosed. However, current clinical practice guidelines recommend starting a BTA in the vast majority of patients irrespective of symptoms [14].

The cross-sectional nature of the study requires a cautious interpretation of our findings. The PROs highlight a snapshot of a single time point on the individual patient’s illness trajectory. It is therefore not possible to deduce or anticipate the evolvement of pain or QoL over time, also because we did not control for the time since BTA treatment start. Randomized trials incorporating repeated pain and QoL measurements showed a delay of worsening rather than an improvement in these outcomes when treated with denosumab compared to zoledronic acid [7]. Local external beam radiotherapy is effective and provides a rapid relief for painful bone metastases [24]. Among the patients with bone complications at diagnosis (*n* = 135), 76% received radiotherapy. We did not control for the time since diagnosis and can therefore not estimate the effect of radiotherapy on the relationship between BTA treatment, pain and quality of life.

Our sample may have been overrepresented with patients who were at an early stage of BTA treatment, although some measures were taken to minimize potential sources of selection within the patient and physician populations. The 3-month study period was determined and informed by current clinical practice guidelines for BTAs and commonly used chemotherapeutic agents to ensure inclusion of all patients with bone metastases, regardless of BTA treatment. The observational nature of the study ensured that findings reflect patients’ experiences while receiving treatment in real-world practice.

In addition to a pain severity measure, we used the FACT-BP, a QoL measure specific to cancer patients with bone metastases. The only other validated tool for this subgroup of patient is EORTC QLQ-BM22 [25]. The choice of the FACT-BP allowed to focus on all emotional and functional items due to bone pain exclusively, while the QLQ-BM22 assess these based on patients’ illness in general [12]. The more concise 16-item FACT-BP may prove beneficial when the impact solely of bone pain on QoL is of interest [12]. The QLQ-BM22 examines bone pain in five main areas, and differentiates between constant and intermittent pain [25], something we did not capture in this study. Although patients were specifically instructed to evaluate expectations and satisfaction with their BTA treatment, it may have been difficult for them to isolate their estimation from the effects of other treatments they received.

In conclusion, PROs support the findings based on the physicians’ perspective suggesting high levels of pain control [11]. Overall, pain and QoL did not significantly differ according to BTA treatment or physicians’ risk perception. Although differences in QoL between patients considered to be at ‘low’ and ‘high’ risk may be a consequence of varying disease burden, there may be a subgroup of patients who may benefit from not starting BTA treatment at diagnosis of bone metastases. Due to the lack of prospective data on factors or characteristics that would justify forgoing BTA treatment, the individual decision of not treating a patient as observed in real-world practice cannot be recommended. However, real world data may add further information for patient care, in particular for the management of subgroups of patients not sufficiently covered by guidelines.

## Supplementary Information

**Additional file 1: Table S1**. Patient characteristics and treatment questionnaire. **Table S2**: Bone pain (FACT-BP) distribution of answers to single items. **Table S3**. QoL scores by pain severity. **Table S4**. Patient vs. physician reported pain scores. **Table S5**. Use of analgesic medication according to patient-reported BPI worst pain scores (N=399). **Table S6**. Pain and QoL scores according to BTA treatment and risk status. **Table S7** Satisfaction with BTA treatment (FACT-TS-G): Distribution of answers to single questions.

## Data Availability

The datasets used and/or analysed during the current study available from the corresponding author on reasonable request.

## Abbreviations

AQA: Analgesic Quantification Algorithm
BPI-SF: Brief Pain Inventory-Short Form
BTAs: Bone-targeted agents
EORTC QLQ-BM22: European Organisation for Research and Treatment of Cancer Quality of Life Questionnaire for patients with Bone Metastases
FACT-G: Functional Assessment of Cancer Therapy – General
FACT-BP: Functional Assessment of Cancer Therapy Bone-pain module
FACIT-TS-G: Functional Assessment of Chronic Illness and Treatment – Treatment Satisfaction-General
PREs: Patient-reported experiences
PROs: Patient-reported outcomes
QoL: Quality of life
RCTs: Randomized clinical trials
SAKK: Swiss Group for Clinical Cancer Research
SREs: Skeletal-related events
SSEs: Symptomatic skeletal events
