# Supplementary material for: Quality of life and pain in patients with metastatic bone disease from solid tumors treated with bone-targeted agents– a real-world cross-sectional study from Switzerland (SAKK 95/16)
Source: BMC Cancer. 2021 Feb 19;21:182. doi: 10.1186/s12885-021-07903-8 (PMC7893880; doi:10.1186/s12885-021-07903-8)
Supplement: Supplementary file 1 — Additional file 1: Table S1. Patient characteristics and treatment questionnaire. Table S2: Bone pain (FACT-BP) distribution of answers to single items. Table S3. QoL scores by pain severity. Table S4. Patient vs. physician reported pain scores. Table S5. Use of analgesic medication according to patient-reported BPI worst pain scores (N=399). Table S6. Pain and QoL scores according to BTA treatment and risk status. Table S7 Satisfaction with BTA treatment (FACT-TS-G): Distribution of answers to single questions. [file 12885_2021_7903_MOESM1_ESM.docx]

**SAKK 9516 Supplementary material**

**Table S1. Patient Characteristics and Treatment questionnaire**

**PART 1: PATIENT KEY CHARACTERISTICS**

1. **What is the patients age and year of birth?**

|  |  |
| --- | --- |
| **Age** | |

|  |  |  |  |
| --- | --- | --- | --- |
| **Year of birth** | | | |

1. **What is the patients highest level of education?**

| ⭘ | Compulsory schooling |
| --- | --- |
| ⭘ | High school degree |
| ⭘ | University degree |
| ⭘ | Vocational school degree (apprenticeship) |
| ⭘ | Unknown |

1. **What is the patients employment/working status?**

| ⭘ | Retired |
| --- | --- |
| ⭘ | Student |
| ⭘ | Unemployed |
| ⭘ | Working part time |
| ⭘ | Working full time |
| ⭘ | Unknown |

1. **Is the patient not able to work as a result of the cancer?**

| ⭘ | Yes |
| --- | --- |
| ⭘ | No |
| ⭘ | Unknown |

- 1. **If yes, for how long has the patient not been able to work**

|  | Years |  | Months | ⭘ Unknown |
| --- | --- | --- | --- | --- |

1. **What is the smoking status of the patient?**

| ⭘ | Current smoker |
| --- | --- |
| ⭘ | Ex-smoker |
| ⭘ | Never smoker |
| ⭘ | Unknown |

1. **What is the underlying tumor entity of the patient?**

| ⭘ | Breast cancer  Please indicate if: ⭘ Endocrine-responsive  ⭘ Endocrine-non-responsive |
| --- | --- |
| ⭘ | Lung cancer |
| ⭘ | Prostate cancer  Please indicate if: ⭘ Castration-resistant  ⭘ Hormone sensitive |
| ⭘ | Other, please specify: _____________________________________ |

1. **When was the patient diagnosed with bone metastases?**

|  |  |  |  |  |  |
| --- | --- | --- | --- | --- | --- |
| **Month** | | **Year** | | | |

1. **Which imaging methods were used for the diagnosis of the patient’s bone metastases?** *(Select all that apply)*

|  | Computerised Tomography (CT) scan |
| --- | --- |
|  | Magnetic Resonance Imaging (MRI) scan |
|  | Positron Emission Tomograhy (PET)-CT |
|  | Scintigraphy |
|  | X-ray |

1. **What are the patient’s current symptoms?** *(Select all that apply)*

|  | Anxiety/Depression |
| --- | --- |
|  | Bone fracture |
|  | Bone pain |
|  | Cough |
|  | Dyspnoe |
|  | Fatigue |
|  | Weight loss |
|  | No symptoms |

1. **What are the patient’s co-morbidities?** *(Select all that apply)*

|  | Cerebrovascular disease |
| --- | --- |
|  | Congestive heart failure |
|  | Connective tissue disease |
|  | COPD |
|  | Coronary heart disease |
|  | Diabetes |
|  | GI disease |
|  | Hypertension |
|  | Liver disease |
|  | Osteoarthritis |
|  | Osteoporosis |
|  | Peripheral vascular disease |
|  | Psychiatric disorders |
|  | Renal impairment |
|  | No underlying conditions |

1. **Please indicate, if laboratory values listed below are assessed?**

|  | **Yes** | **No** |
| --- | --- | --- |
| Albumin | ⭘ | ⭘ |
| Alkaline phosphatase | ⭘ | ⭘ |
| Calcium | ⭘ | ⭘ |
| Calcium corrected | ⭘ | ⭘ |
| Creatinine | ⭘ | ⭘ |
| Vitamin D | ⭘ | ⭘ |

1. **What is the current ECOG status of the patient?**

| ⭘ | **0** | Fully active, able to carry on all pre-disease performance without restriction |
| --- | --- | --- |
| ⭘ | **1** | Restricted in physically strenuous activity but ambulatory and able to carry out light work |
| ⭘ | **2** | Ambulatory and capable of all self-care but unable to carry out work activities. Up and about more than 50% of waking hours |
| ⭘ | **3** | Capable of only limited self-care, confined to bed or chair more than 50% of waking hours |
| ⭘ | **4** | Completely disabled. Cannot carry on any self-care. Confined to bed or chair |

1. **Has the patient ever received one of the following cancer treatments?** *(Select all that apply)*

|  | Chemotherapy |
| --- | --- |
|  | Hormone therapy |
|  | Immunotherapy |
|  | Radiotherapy |
|  | Radioisotope therapy |
|  | Surgery |
|  | Targeted treatments |

1. **Does the patient experience any side effects from the current cancer treatment?**

| ⭘ | Yes |
| --- | --- |
| ⭘ | No |

1. **What is the current disease status of the patient?**

| ⭘ | Progressing |
| --- | --- |
| ⭘ | Stable |

1. **Please select all supportive therapies currently received by the patient:** *(Select all that apply)*

|  | Anti-depressants |
| --- | --- |
|  | Anti-emetics |
|  | Corticosteroids |
|  | Non-opioid analgesics |
|  | Opioid analgesics |

1. **Please indicate all sites of current metastases?** *(Select all that apply)*

|  | Bone |
| --- | --- |
|  | Brain |
|  | Liver |
|  | Lung |
|  | Lymph nodes |
|  | Other |

1. **What is the current number of bone metastases?**

| ⭘ | < 3 bone metastases |
| --- | --- |
| ⭘ | ≥ 3 bone metastases |

1. **Was the patient experiencing bone pain at diagnosis of bone metastases?**

| ⭘ | No bone pain |
| --- | --- |
| ⭘ | Mild bone pain |
| ⭘ | Moderate bone pain |
| ⭘ | Severe bone pain |
| ⭘ | Not assessed |

1. **Is the patient currently experiencing bone pain due to bone metastases?**

| ⭘ | No bone pain |
| --- | --- |
| ⭘ | Mild bone pain |
| ⭘ | Moderate bone pain |
| ⭘ | Severe bone pain |
| ⭘ | Not assessed |

1. **How is the patient’s current bone pain treated according to the analgesic quantification algorithm (AQA) score?**

| ⭘ | 0 = no analgesic use |
| --- | --- |
| ⭘ | 1 = non-opioid analgesics |
| ⭘ | 2 = weak opioids |
| ⭘ | 3 = strong opioids ≤ 75 mg OME* per day |
| ⭘ | 4 = strong opioids > 75-150 mg OME per day |
| ⭘ | 5 = strong opioids > 150-300 mg OME per day |
| ⭘ | 6 = strong opioids > 300-600 mg OME per day |
| ⭘ | 7 = strong opioids > 600 mg OME per day |

* **OME** = Oral Morphine Equivalent

1. **Did the patient experience any complications at diagnosis of bone metastases?**

(*such as pathologic fractures, spinal cord compression, bone radiation or bone surgery*)

| ⭘ | Yes |
| --- | --- |
| ⭘ | No |

- 1. **If yes, which of the following complications did the patient experience?** *(Select all that apply)*

|  | Bone radiation |
| --- | --- |
|  | Bone surgery |
|  | Hypercalcaemia |
|  | Pathologic fracture |
|  | Spinal cord compression |
|  | Other bone complications |

1. **Is the patient currently experiencing any bone complications?**

(*such as pathologic fractures, spinal cord compression, bone radiation or bone surgery*)

| ⭘ | Yes |
| --- | --- |
| ⭘ | No |

- 1. **If yes, which of the following complications is the patient currently experiencing?** *(Select all that apply)*

|  | Bone radiation |
| --- | --- |
|  | Bone surgery |
|  | Hypercalcaemia |
|  | Pathologic fracture |
|  | Spinal cord compression |
|  | Other bone complications |

1. **Where were the bone metastases located at diagnosis?** *(Select all that apply)*

|  | Arm |
| --- | --- |
|  | Hip/Pelvis |
|  | Leg |
|  | Ribs |
|  | Skull |
|  | Vertebrae |

1. **Has the patient ever been treated with bone-targeted agents (BTA) after the diagnosis of bone metastases?**

| ⭘ | Yes |
| --- | --- |
| ⭘ | No |

- 1. **If no, why was the patient NOT treated with BTA ?**

*Please rank your top 3 reasons with 1-3 (1 = the most important)*

| ______ | Costs |
| --- | --- |
| ______ | Focus on treating the primary tumor |
| ______ | Pill burden |
| ______ | Low risk of bone complications |
| ______ | Patient refusal |
| ______ | Poor performance status |
| ______ | Poor renal function |
| ______ | Risk of osteonecrosis of the jaw (ONJ) |
| ______ | Risk of hypocalcaemia |
| ______ | Short life expectancy |
| ______ | Very recent diagnosis, so no time to initiate |

- - 1. **If you selected risk of ONJ as one of your top reasons for delaying the initiation of BTA why do you believe that there is particular risk of ONJ for this patient?** *(Select all that apply)*

|  | Antiangiogenics |
| --- | --- |
|  | Chemotherapy agents |
|  | Diabetes mellitus |
|  | Immunosuppressants |
|  | Jaw pain |
|  | Renal function |
|  | Tooth extraction |
|  | Systemic corticosteroids |

- - 1. **If you selected patient refusal as one of your top reasons for delaying the initiation of BTA for what reason did the patient refuse bone targeted therapy?**

| ⭘ | Afraid of side effects |
| --- | --- |
| ⭘ | Tired of treatment |
| ⭘ | Other |

1. **Has the patient received any pre-treatment for osteoporosis ?** *(Select all that apply)*

|  | Bisphosphonates |
| --- | --- |
|  | Calcium/Vitamin D |
|  | Denosumab |
|  | None |

**PART 2: PATIENT CONSULTATION**

1. **Since when do you manage the patient’s cancer?**

|  |  |  |  |  |  |
| --- | --- | --- | --- | --- | --- |
| **Month** | | **Year** | | | |

1. **How regular do you see the patient ?** *(Select most applicable)*

| ⭘ | Once per year |
| --- | --- |
| ⭘ | Once per month |
| ⭘ | Once per week |

1. **How often was the patient hospitalized due to the bone metastases?** *(Please enter a number)*

|  | **OR** | ⭘ Unknown |
| --- | --- | --- |

1. **What is the patient’s current life expectancy?** *(Select most applicable)*

| ⭘ | Weeks |
| --- | --- |
| ⭘ | Months |
| ⭘ | Years |

**PART 3: TO BE COMPLETED ONLY IF PATIENT IS TREATED WITH BONE-TARGETING AGENTS (BTA)**

1. **Please provide the date the patient first received BTA?**

|  |  |  |  |  |  |
| --- | --- | --- | --- | --- | --- |
| **Month** | | **Year** | | | |

1. **Please provide the main reasons why you have treated the patient with BTA at diagnosis of bone metastases:** *Please rank your top 3 reasons with 1-3 (1 = the most important)*

| ______ | Bone pain |
| --- | --- |
| ______ | Good performance status |
| ______ | High risk of bone complications |
| ______ | Location of bone metastases |
| ______ | Long life expectancy |
| ______ | Number of bone metastases |
| ______ | Patient’s request |
| ______ | Prior history of bone complications |

1. **Which BTA did the patient receive at diagnosis of bone metastases?**

| ⭘ | Denosumab |
| --- | --- |
| ⭘ | Ibandronate |
| ⭘ | Pamidronic acid |
| ⭘ | Zoledronic acid |

- 1. **Please provide the main reasons why you have treated the patient with the BTA chosen in question 3:** *Please rank your top 3 reasons with 1-3 (1 = the most important)*

| ____ | Costs |
| --- | --- |
| ____ | Efficacy |
| ____ | Hospital/local standards |
| ____ | Lower risk of toxicities |
| ____ | Mode of administration |
| ____ | Familiarity with the treatment |
| ____ | Patient’s preference |

- 1. **Please provide the total number of BTA doses the patient received and the treatment length:**

Number of doses: ___________

Treatment length in months: ___________

1. **Are markers for bone remodelling (e.g. uNTx) assessed regularly?**

| ⭘ | Yes |
| --- | --- |
| ⭘ | No |

1. **Are calcium & albumin levels measured?** *(Select most applicable)*

| ⭘ | Yes, before every dose of BTA |
| --- | --- |
| ⭘ | Yes, after every dose of BTA |
| ⭘ | Occasionally/irregularly |
| ⭘ | No |

1. **Was the patient pre-treated with calcium/vitamin D supplementation?**

| ⭘ | Yes, before first dose of BTA |
| --- | --- |
| ⭘ | Yes, after first dose of BTA |
| ⭘ | No |

1. **Please indicate your experience with calcium/vitamin D supplementation.**

Calcium

| ⭘ | Well tolerated by patient |
| --- | --- |
| ⭘ | Not well tolerated by patient |

Vitamin D

| ⭘ | Well tolerated by patient |
| --- | --- |
| ⭘ | Not well tolerated by patient |

- 1. **If either calcium or vitamin D was NOT well tolerated, was the product ever changed?**

| ⭘ | Yes |
| --- | --- |
| ⭘ | No |

1. **Please indicate the dosing interval you prescribed for BTA administration:**

| ⭘ | Every 3-4 weeks |
| --- | --- |
| ⭘ | Every 3-4 weeks unless there is a substantial fall in the patients performance status |
| ⭘ | Every 3-4 weeks for 3 months and then once every 12 weeks |
| ⭘ | Every 3-4 weeks for 1 year and then once every 12 weeks |
| ⭘ | Every 3-4 weeks for 2 years and then once every 12 weeks |
| ⭘ | Every 12 weeks |
| ⭘ | Every 24 weeks |

1. **Please provide the main reasons for prescribing this dosing interval:**

*Please rank your top 3 reasons with 1-3 (1 = the most important).*

| ____ | Costs |
| --- | --- |
| ____ | Most convenient for patient |
| ____ | Most clinical benefit |
| ____ | Most convenient with patient’s cancer treatment schedule |
| ____ | New data available for a specific dosing interval |

1. **Was the dosing interval of BTA changed during the course of treatment?**

| ⭘ | Yes, temporarily |
| --- | --- |
| ⭘ | Yes, permanently |
| ⭘ | No |

- 1. **If yes, please indicate the dosing interval following the change:**

| ⭘ | Every 3-4 weeks |
| --- | --- |
| ⭘ | Every 3-4 weeks unless there is a substantial fall in the patients performance status |
| ⭘ | Every 3-4 weeks for 3 months and then once every 12 weeks |
| ⭘ | Every 3-4 weeks for 1 year and then once every 12 weeks |
| ⭘ | Every 3-4 weeks for 2 years and then once every 12 weeks |
| ⭘ | Every 12 weeks |
| ⭘ | Every 24 weeks |

- 1. **If yes, when was the dosing interval changed?**

| ⭘ | 0-3 months after initiation of BTA |
| --- | --- |
| ⭘ | 4-6 months after initiation of BTA |
| ⭘ | 7-12 months after initiation of BTA |
| ⭘ | 13-18 months after initiation of BTA |
| ⭘ | 19-24 months after initiation of BTA |
| ⭘ | >24 months after initiation of BTA |

- 1. **If yes, please provide the main reasons for changing the previous dosing interval.** (*Select all that apply*):

|  | Allergic reaction |
| --- | --- |
|  | Patient’s preference |
|  | Poor renal function |
|  | Presence of bone complications |
|  | Presence of osteonecrosis of the jaw (ONJ) |
|  | Presence of hypocalcaemia |
|  | Synchronization with other therapies (i.e. chemotherapy) |

1. **Is the patient still receiving the BTA selected in question 3?**

| ⭘ | Yes |
| --- | --- |
| ⭘ | No |

- 1. **If no, date treatment stopped**

|  |  |  |  |  |  |  |  |
| --- | --- | --- | --- | --- | --- | --- | --- |
| **Day** | | **Month** | | **Year** | | | |

- 1. **Please indicate why the patient is no longer treated with the BTA selected in question 3:**

| ⭘ | Patient discontinued the treatment | **go to question 12** |
| --- | --- | --- |
| ⭘ | Patient switched to another BTA | **go to question 13** |

1. **Please provide the main reasons why the patient discontinued BTA treatment:**

*Please rank your top 3 reasons with 1-3 (1 = the most important).*

| ____ | Costs |
| --- | --- |
| ____ | Development of bone complications |
| ____ | Disease progression |
| ____ | End of planned treatment |
| ____ | Hypocalcaemia |
| ____ | Lack of patient compliance |
| ____ | Patient’s request |
| ____ | Poor performance status |
| ____ | Poor renal function |
| ____ | Presence of Osteonecrosis of the jar (ONJ) |
| ____ | Short life expectancy |

- 1. **If bone complications were selected above, please specify:** *(Select all that apply)*

|  | Bone radiation |
| --- | --- |
|  | Bone surgery |
|  | Change in antineoplastic treatment |
|  | Hypercalcaemia |
|  | Pathologic fracture |
|  | Spinal cord compression |
|  | Other bone complications |

1. **Please indicate to which other BTA treatment the patient has been switched to:**

| ⭘ | Denosumab |
| --- | --- |
| ⭘ | Ibandronate |
| ⭘ | Pamidronic acid |
| ⭘ | Zoledronic acid |

- 1. **Please provide the main reasons why the patient was switched to this BTA:**

*Please rank your top 3 reasons with 1-3 (1 = the most important).*

| ____ | Costs |
| --- | --- |
| ____ | Efficacy |
| ____ | Hospital/local standards |
| ____ | Lower risk of toxicities |
| ____ | Mode of administration |
| ____ | Familiarity with the treatment |
| ____ | Patient’s preference |

1. **Is the patient currently receiving the BTA switched to?**

| ⭘ | Yes |
| --- | --- |
| ⭘ | No |

- 1. **If yes, please provide the total number of BTA doses the patient received and the treatment length:**

Number of doses: ___________

Treatment length in months: ___________

1. **Please indicate the dosing interval you prescribed for the BTA selected in question 13:**

| ⭘ | Every 3-4 weeks |
| --- | --- |
| ⭘ | Every 3-4 weeks unless there is a substantial fall in the patients performance status |
| ⭘ | Every 3-4 weeks for 3 months and then once every 12 weeks |
| ⭘ | Every 3-4 weeks for 1 year and then once every 12 weeks |
| ⭘ | Every 3-4 weeks for 2 years and then once every 12 weeks |
| ⭘ | Every 12 weeks |
| ⭘ | Every 24 weeks |

1. **What is the main reason for prescribing this dosing interval?**

*Please rank your top 3 reasons with 1-3 (1 = the most important).*

| ____ | Costs |
| --- | --- |
| ____ | Most convenient for patient |
| ____ | Most clinical benefit |
| ____ | Most convenient with patient’s cancer treatment schedule |
| ____ | New data available for a specific dosing interval |

**Table S2: Bone pain (FACT-BP) distribution of answers to single items**

| **I have certain parts of my body where I experience pain** | | | |
| --- | --- | --- | --- |
|  | N | Percent | |
| 0 | 99 | 23.91 | |
| 1 | 107 | 25.85 | |
| 2 | 67 | 16.18 | |
| 3 | 39 | 9.42 | |
| 4 | 24 | 5.80 | |
| Missing | 78 | 18.84 | |
| **I am content with the quality of my life right now** |  |  | |
|  | N | Percent | |
| Not at all | 27 | 6.52 | |
| A little bit | 22 | 5.31 | |
| Somewhat | 112 | 27.05 | |
| Quite a bit | 169 | 40.82 | |
| Very much | 72 | 17.39 | |
| Missing | 12 | 2.90 | |
| **I have certain body parts where I experience pain** |  |  | |
|  | N | Percent | |
| Not at all | 148 | 35.75 | |
| A little bit | 110 | 26.57 | |
| Somewhat | 73 | 17.63 | |
| Quite a bit | 42 | 10.14 | |
| Very much | 21 | 5.07 | |
| Missing | 20 | 4.83 | |
| **I have bone pain** | | | |
|  | N | | Percent |
| Not at all | 173 | | 41.79 |
| A little bit | 105 | | 25.36 |
| Somewhat | 71 | | 17.15 |
| Quite a bit | 34 | | 8.21 |
| Very much | 19 | | 4.59 |
| Missing | 12 | | 2.90 |
| **It hurts when I put weight or pressure on the place where I have bone pain** | | | |
|  | N | | Percent |
| Not at all | 201 | | 48.55 |
| A little bit | 86 | | 20.77 |
| Somewhat | 52 | | 12.56 |
| Quite a bit | 38 | | 9.18 |
| Very much | 23 | | 5.56 |
| Missing | 14 | | 3.38 |

**Table S2 continued**

| **I have bone pain even when I sit or lie still** | | | |
| --- | --- | --- | --- |
|  | N | | Percent |
| Not at all | 254 | | 61.35 |
| A little bit | 72 | | 17.39 |
| Somewhat | 44 | | 10.63 |
| Quite a bit | 25 | | 6.04 |
| Very much | 11 | | 2.66 |
| Missing | 8 | | 1.93 |
| **I need help doing my usual activities because of bone pain** | | | |
|  | | N | Percent |
| Not at all | | 296 | 71.50 |
| A little bit | | 54 | 13.04 |
| Somewhat | | 27 | 6.52 |
| Quite a bit | | 11 | 2.66 |
| Very much | | 14 | 3.38 |
| Missing | | 12 | 2.90 |
| **I am forced to rest during the day because of bone pain** | | | |
|  | | N | Percent |
| Not at all | | 249 | 60.14 |
| A little bit | | 68 | 16.43 |
| Somewhat | | 43 | 10.39 |
| Quite a bit | | 27 | 6.52 |
| Very much | | 18 | 4.35 |
| Missing | | 9 | 2.17 |
| **I have trouble walking because of bone pain** | | | |
|  | | N | Percent |
| Not at all | | 239 | 57.73 |
| A little bit | | 83 | 20.05 |
| Somewhat | | 33 | 7.97 |
| Quite a bit | | 28 | 6.76 |
| Very much | | 21 | 5.07 |
| Missing | | 10 | 2.42 |
| **Bone pain interferes with my ability to care for myself (bathing, dressing, eating, etc.)** | | | |
|  | | N | Percent |
| Not at all | | 322 | 77.78 |
| A little bit | | 34 | 8.21 |
| Somewhat | | 27 | 6.52 |
| Quite a bit | | 12 | 2.90 |
| Very much | | 9 | 2.17 |
| Missing | | 10 | 2.42 |
| **Bone pain interferes with my social activities** | | | |
|  | | N | Percent |
| Not at all | | 260 | 62.80 |
| A little bit | | 68 | 16.43 |
| Somewhat | | 35 | 8.45 |
| Quite a bit | | 25 | 6.04 |
| Very much | | 18 | 4.35 |
| Missing | | 8 | 1.93 |

**Table S2 continued**

| **Bone pain wakes me up at night** | | |
| --- | --- | --- |
|  | N | Percent |
| Not at all | 289 | 69.81 |
| A little bit | 68 | 16.43 |
| Somewhat | 27 | 6.52 |
| Quite a bit | 12 | 2.90 |
| Very much | 9 | 2.17 |
| Missing | 9 | 2.17 |
| **I am frustrated by my bone pain** | | |
|  | N | Percent |
| Not at all | 267 | 64.49 |
| A little bit | 68 | 16.43 |
| Somewhat | 30 | 7.25 |
| Quite a bit | 20 | 4.93 |
| Very much | 17 | 4.11 |
| Missing | 12 | 2.90 |
| **I feel depressed about my bone pain** | | |
|  | N | Percent |
| Not at all | 289 | 69.81 |
| A little bit | 59 | 14.25 |
| Somewhat | 27 | 6.52 |
| Quite a bit | 15 | 3.62 |
| Very much | 13 | 3.14 |
| Missing | 11 | 2.66 |
| **I worry that my bone pain will get worse** | | |
|  | N | Percent |
| Not at all | 183 | 44.20 |
| A little bit | 108 | 26.09 |
| Somewhat | 44 | 10.63 |
| Quite a bit | 39 | 9.42 |
| Very much | 31 | 7.49 |
| Missing | 9 | 2.17 |
| **My family has trouble understanding when my bone pain interferes with my activity** | | |
|  | N | Percent |
| Not at all | 312 | 73.36 |
| A little bit | 50 | 12.08 |
| Somewhat | 16 | 3.86 |
| Quite a bit | 10 | 2.42 |
| Very much | 7 | 1.69 |
| Missing | 19 | 4.59 |

**Table S3. QoL scores by pain severity**

|  | **No / mild pain**  (BPI 0-4) | | | **Moderate / severe pain**  (BPI 5-10) | | |  |  |
| --- | --- | --- | --- | --- | --- | --- | --- | --- |
|  | **N** | **Mean** | **SD** | **N** | **Mean** | **SD** | **95% CI for difference^2^** | ***p* value^3^** |
| **Bone pain (FACT-BP)^1^** | 286 | 53.2 | 7.0 | 113 | 36.7 | 13.9 | (13.0, 18.9) | <0.001 |
| **Quality of Life (FACT-G)^1^** |  |  |  |  |  |  |  |  |
| Physical wellbeing | 286 | 22.6 | 4.3 | 113 | 16.1 | 6.0 | (5.0, 8.0) | <0.001 |
| Social/family wellbeing | 283 | 22.1 | 5.1 | 114 | 21.3 | 4.8 | (0.0, 2.0) | 0.040 |
| Emotional wellbeing | 285 | 18.3 | 4.3 | 113 | 16.4 | 4.8 | (1.0, 3.0) | <0.001 |
| Functional wellbeing | 286 | 19.1 | 5.3 | 114 | 15.6 | 4.6 | (3.0, 5.0) | <0.001 |
| FACT-G total score | 282 | 82.2 | 14.2 | 112 | 69.3 | 13.9 | (10.2, 16.7) | <0.001 |

^1^ higher scores indicate less bone pain or better QoL

^2^ Hodges–Lehmann estimator

^3^Univariate Wilcoxon–Mann–Whitney tests

**Table S4. Patient vs. physician reported pain scores**

|  | **BPI worst pain (N=400)** | | | | **FACT Bone pain (N=406)** | | |
| --- | --- | --- | --- | --- | --- | --- | --- |
|  | No pain (0)  N | Mild pain (1-4)  N | Moderate pain  (5-6)  N | Severe pain (7-10)  N | N | Mean | SD |
| **Physicians’ estimation** |  |  |  |  |  |  |  |
| No Pain | 95 | 89 | 17 | 10 | 214 | 53.9 | 7.6 |
| Mild pain | 15 | 66 | 24 | 22 | 128 | 45.2 | 11.6 |
| Moderate Pain | 2 | 15 | 12 | 15 | 46 | 39.0 | 12.9 |
| Severe Pain | 0 | 4 | 2 | 12 | 18 | 31.1 | 16.7 |
| Total | 112 | 174 | 55 | 59 |  |  |  |

**Note:** For 17 patients BPI scores were missing, for 11 patients FACT Bone pain was missing

**Table S5. Use of analgesic medication according to patient-reported BPI worst pain scores (N=399)**

|  | **BPI worst pain** | | | |
| --- | --- | --- | --- | --- |
|  | No pain (0)  N (%) | Mild pain (1-4)  N (%) | Moderate pain (5-6)  N (%) | Severe pain (7-10)  N (%) |
| **AQA score** |  |  |  |  |
| 0 (no analgesics) | 84 (44.2) | 84 (44.2) | 17 (9.0) | 5 (2.6) |
| 1-2 (non-opioid analgesics) | 21 (14.7) | 69 (48.3) | 28 (19.6) | 25 (17.5) |
| 3-7 (strong opioids) | 7 (10.6) | 20 (30.3) | 10 (15.2) | 29 (43.9) |
| Total | 112 | 173 | 55 | 59 |

**Note:** AQA= Analgesic Quantification Algorithm; for 1 patients AQA score was missing, for 17 patients BPI scores were missing

**Table S6. Pain and QoL scores according to BTA treatment and risk status**

|  | **Treated**  **High risk** | | |  | **Treated**  **Low risk** | | | **Untreated**  **High risk** | | | | **Untreated**  **Low risk** | | |  |
| --- | --- | --- | --- | --- | --- | --- | --- | --- | --- | --- | --- | --- | --- | --- | --- |
|  | **N** | **Mean** | **SD** |  | **N** | **Mean** | **SD** | **N** | **Mean** | **SD** |  | **N** | **Mean** | **SD** | ***p* value^3^** |
| **Pain (BPI)^1^** |  |  |  |  |  |  |  |  |  |  |  |  |  |  |  |
| Worst pain | 190 | 3.0 | 2.9 |  | 93 | 3.4 | 2.8 | 39 | 3.4 | 3.1 |  | 62 | 2.0 | 2.3 | 0.025 |
| Least pain | 189 | 1.1 | 1.7 |  | 93 | 1.4 | 1.6 | 39 | 1.2 | 1.6 |  | 62 | 1.0 | 1.6 | 0.349 |
| Average pain | 190 | 2.0 | 2.0 |  | 93 | 2.3 | 2.1 | 39 | 2.4 | 2.5 |  | 62 | 1.6 | 1.8 | 0.218 |
| Pain right now | 190 | 1.5 | 2.1 |  | 93 | 2.0 | 2.3 | 39 | 1.6 | 2.1 |  | 62 | 1.2 | 1.9 | 0.107 |
| **Bone pain (FACT-BP)^2^** | 191 | 48.0 | 12.0 |  | 97 | 46.9 | 12.6 | 39 | 48.5 | 12.5 |  | 63 | 52.0 | 10.1 | 0.012 |
| **Quality of Life (FACT-G)^2^** |  |  |  |  |  |  |  |  |  |  |  |  |  |  |  |
| Physical wellbeing | 192 | 20.8 | 5.8 |  | 97 | 19.7 | 5.8 | 39 | 19.7 | 5.8 |  | 63 | 22.4 | 5.1 | 0.011 |
| Social/family wellbeing | 192 | 21.6 | 5.2 |  | 95 | 21.8 | 4.9 | 39 | 23.6 | 3.7 |  | 63 | 22.3 | 4.6 | 0.171 |
| Emotional wellbeing | 192 | 17.6 | 4.8 |  | 95 | 17.0 | 4.7 | 39 | 18.4 | 4.6 |  | 63 | 18.5 | 3.9 | 0.231 |
| Functional wellbeing | 193 | 17.7 | 5.6 |  | 97 | 17.7 | 5.0 | 39 | 17.5 | 5.5 |  | 63 | 19.6 | 4.8 | 0.093 |
| FACT-G total score | 190 | 77.8 | 15.5 |  | 94 | 76.1 | 15.9 | 39 | 79.2 | 14.8 |  | 63 | 82.8 | 14.2 | 0.072 |

^1^ higher score indicate worse pain

^2^ higher scores indicate less bone pain or better QoL

^3^ Univariate Kruskal-Wallis tests

**Table S7 Satisfaction with BTA treatment (FACT-TS-G): Distribution of answers
to single questions**

| **Compared to what you expected, how do you rate the effectiveness of the treatment so far?** | | |
| --- | --- | --- |
|  | N | Percent |
| A lot worse | 6 | 2.24 |
| A little worse | 9 | 3.36 |
| About the same | 73 | 27.24 |
| A little better | 48 | 17.91 |
| A lot better | 99 | 36.94 |
| Missing | 33 | 12.31 |
| **Compared to what you expected, how do you rate the side effects of treatment so far?** | | |
|  | N | Percent |
| A lot worse | 10 | 3.73 |
| A little worse | 33 | 12.31 |
| About the same | 80 | 29.85 |
| A little better | 35 | 13.06 |
| A lot better | 76 | 28.36 |
| Missing | 34 | 12.69 |
| **Did your doctor(s) help you evaluate the effects of your treatment so far?** | | |
|  | N | Percent |
| No, not at all | 5 | 1.87 |
| Yes, to some extent | 19 | 7.09 |
| Yes, for the most part | 83 | 30.97 |
| Yes, completely | 127 | 47.39 |
| Missing | 34 | 12.69 |
| **Do you feel you received the treatment that was right for you?** | | |
|  | N | Percent |
| No, not at all | 3 | 1.12 |
| Yes, to some extent | 16 | 5.97 |
| Yes, for the most part | 67 | 25.00 |
| Yes, completely | 152 | 56.72 |
| Missing | 30 | 11.19 |
| **Are you satisfied with the effects of this treatment so far?** | | |
|  | N | Percent |
| No, not at all | 7 | 2.61 |
| Yes, to some extent | 27 | 10.07 |
| Yes, for the most part | 65 | 24.25 |
| Yes, completely | 133 | 49.63 |
| Missing | 36 | 13.43 |
| **Would you recommend this treatment to others with your illness?** | | |
|  | N | Percent |
| No | 4 | 1.49 |
| Maybe | 60 | 22.39 |
| Yes | 168 | 62.69 |
| Missing | 36 | 13.43 |
| **Would you choose this treatment again?** | | |
|  | N | Percent |
| No | 8 | 2.99 |
| Maybe | 51 | 19.03 |
| Yes | 173 | 64.55 |
| Missing | 36 | 13.43 |

**Table S7 continued**

| **How do you rate this treatment overall?** | | |
| --- | --- | --- |
|  | N | Percent |
| Poor | 2 | 0.75 |
| Fair | 15 | 5.60 |
| Good | 67 | 25.00 |
| Very good | 86 | 32.06 |
| Excellent | 64 | 23.88 |
| Missing | 34 | 12.69 |
